# Bilateral testicular mass in a 21-year-old male with a history of congenital adrenal hyperplasia: A case report

**DOI:** 10.1016/j.eucr.2025.102964

**Published:** 2025-01-29

**Authors:** Amir Reza Abedi, Azade Rakhshan, Mohammad Seifi Poor

**Affiliations:** a Men's Health and Reproductive Health Research Center, Shahid Beheshti University of Medical Sciences, Tehran, Iran; b Department of Pathology, Shohada-e-tajrish Educational Hospital, School of Medicine, Shahid Beheshti University of Medical Sciences, Tehran, Iran; c Department of Urology, Shohada-e-tajrish Educational Hospital, School of Medicine, Shahid Beheshti University of Medical Sciences, Tehran, Iran

## Abstract

A 21-year-old man was referred because of bilateral testicular swelling from 8 months ago. He had a known history of congenital adrenal hyperplasia (21-hydroxylase deficiency) diagnosed in infancy, managed with glucocorticoid therapy since childhood. However, the patient had been non-adherent to medication for several years. The patient underwent surgery with an inguinal incision, and a biopsy from the mass was sent for frozen section. The frozen and permanent sections were consistent with “Testicular adrenal rest tumor (tumor of adrenogenital syndrome)

## 1 Introduction

Congenital adrenal hyperplasia (CAH) is a group of autosomal recessive disorders due to pathogenic variants in genes encoding enzymes and cofactors involved in adrenal steroidogenesis.^1^. 21-hydroxylase deficiency (21-OHD) is the most common cause of adrenal hyperplasia (CAH).^2^. This condition often results in ambiguous genitalia, early virilization, and infertility issues. In rare cases, CAH may predispose individuals to the development of testicular masses, often referred to as adrenal rest tumors, which arise due to ectopic adrenal tissue in the testes.^1^.

## 2 Case presentation

A 21-year-old man was referred to the urology clinic with bilateral testicular swelling from 8 months ago. There was no history of trauma, fever, or recent illness. The patient reported no urinary or sexual dysfunction. He had a known history of congenital adrenal hyperplasia (21-hydroxylase deficiency) diagnosed in infancy, managed with glucocorticoid therapy since childhood. However, the patient had been non-adherent to medication for several years, citing concerns about long-term steroid use and side effects.

On examination, the patient appeared generally healthy but exhibited signs of virilization, including facial hair and increased muscle mass. Both testes were palpable and significantly enlarged, measuring approximately 4 cm bilaterally. The masses were firm, non-tender, and had no overlying skin changes. There were no palpable inguinal lymph nodes.

## 3 Laboratory finding

Serum testosterone: Elevated (consistent with hyperandrogenism).

Serum cortisol: Low (reflecting the underlying CAH and non-adherence to steroid therapy).

Serum adrenocorticotropic hormone (ACTH): Elevated (suggesting adrenal hyperactivity).

Serum tumor markers: Alpha-fetoprotein (AFP), beta-human chorionic gonadotropin (beta-hCG), and lactate dehydrogenase (LDH) were all within normal limits.

## 4 Imaging studies

Scrotal Ultrasound: Bilateral, well-circumscribed hypoechoic masses within the testes, without evidence of calcifications or necrosis (40 × 24 mm and a volume of 20 cc in the right testicle, and a mass with similar characteristics measuring 48 × 32 mm and a volume of 34 cc in the left testicle).

**Fig-1:**
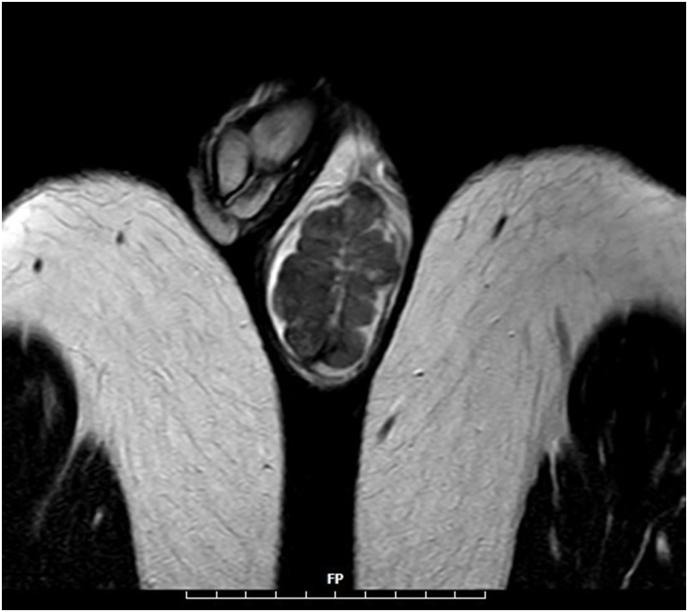
The MRI of testis: Isointense mass on T1-weighted images and hypointense mass on T2-weighted images, relative to the normal parenchyma.

MRI (without contrast): The image shows a mass with lobular margins measuring 42 × 28 mm in the right testicle and another mass measuring 56 × 35 mm in the left testicle. Isointense on T1-weighted images and hypointense on T2-weighted images, relative to the normal parenchyma suggests an adrenal rest tumor (figure-1).

## 5 Management

**Fig-2:**
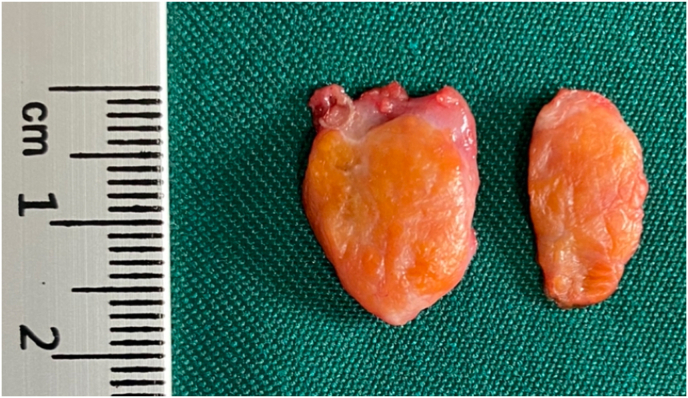
The tumor is non-capsulated and well circumscribed with yellowish brown lobulated cut surface and intervening fibrous bands.

The patient was suspicious for testicular adrenal rest tumors (TART) secondary to poorly controlled congenital adrenal hyperplasia, but we cannot rule out seminoma; therefor, the patient was scheduled for surgery in order to rule out seminoma. The patient underwent surgery with an inguinal incision, and a biopsy from the mass was sent for frozen section. Macroscopic examination revealed two well defined non-capsulated ovoid nodules measuring 2x1.5 × 0.5cm and 2x1x0.4cm with creamy red smooth external surface and brown cut surface with some fine white fibrous bands (Fig. 2).

**Fig-3:**
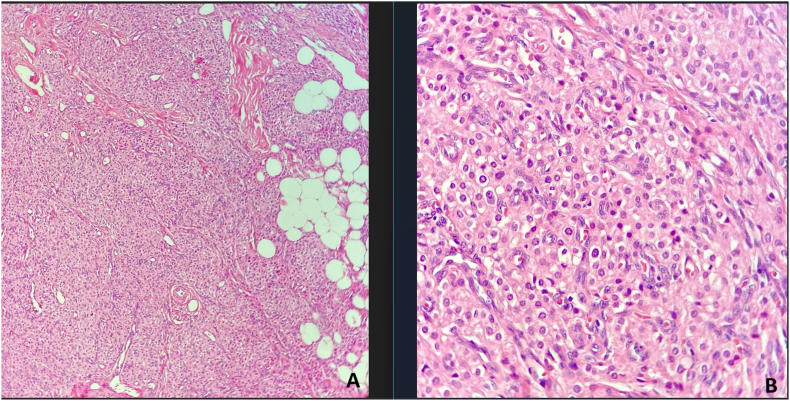
*Tumor consists of lobules of large polygonal cells separated by fibrovascular stroma. Areas of adipocytes metaplasia is present. (Hematoxylin & Eosin stain, x100)* (A)-*Tumoral cells resemble adrenocortical cells with round nuclei and abundant finely granular eosinophilic cytoplasm. Mitosis is absence. (H&E stain, x400) (B)*.

**Fig-4:**
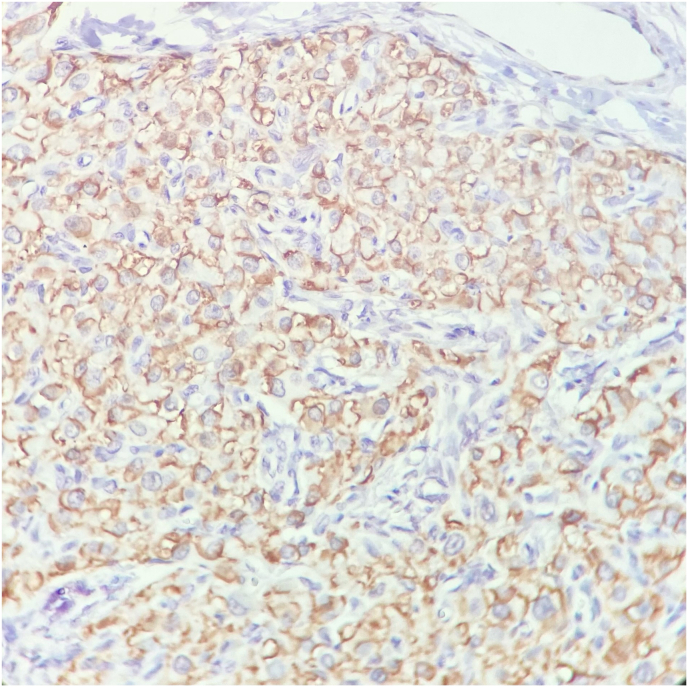
Immunohistochemical staining for inhibin shows cytoplasmic staining of tumoral cells. (x400).

**Fig-5:**
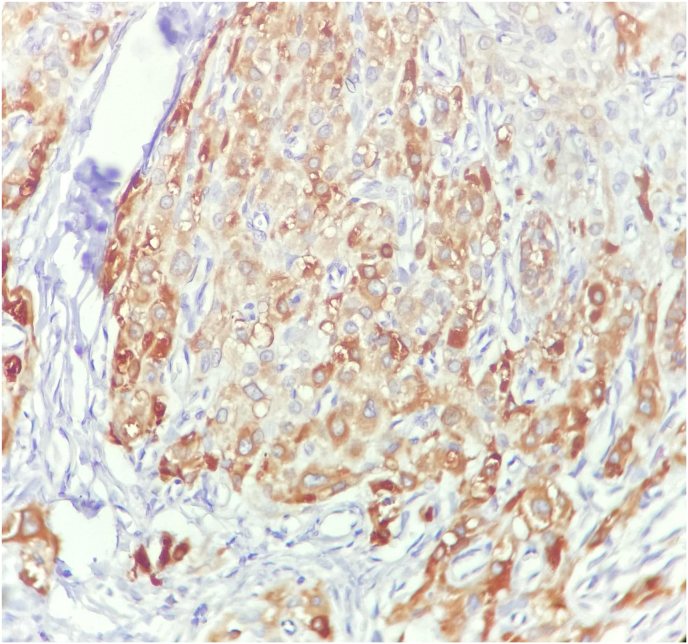
CD56 staining shows diffuse membraneous positivity in tumoral cells. (x400).

In microscopic examination, a neoplasm composed of sheets and lobules of polygonal cells with distinct cell border, round central nuclei, occasional small nucleoli and abundant finely granular pale eosinophilic cytoplasm with scant lipochrome pigment was seen which was reminiscent of adrenocortical cells. Mitosis was not seen. Reinke crystal was absent (Fig. 3). Immunohistochemical (IHC) staining for inhibin and CD-56 was used to help the diagnosis which revealed cytoplasmic staining of tumor cells for inhibin and membranous positivity for CD-56. (Fig-4, Fig-5). Inhibin positivity can be seen in both Leydig cell tumors and TART. Leydig cell tumors are histologically the main differential diagnosis which usually unilateral, may contain Reinke crystals, lack adipocytes metaplasia and extensive fibrosis and are generally negative for neuroendocrine markers such as CD-56 and synaptophysin. For this reason, we used CD-56 for distinction of this tumor from Leydig cell tumor. so the overall histopathologic findings in frozen and permanent sections and IHC study were consistent with “Testicular adrenal rest tumor (tumor of adrenogenital syndrome) “. Based on the pathology report, the testicle was returned to its anatomical location and the surgery was completed. The patient was counseled on the importance of strict adherence to glucocorticoid therapy to suppress ACTH-driven adrenal tissue hyperplasia. He was restarted on a regimen of hydrocortisone, with dose adjustments based on clinical response and hormone levels. Additionally, the potential for fertility preservation was discussed. The primary focus was on medical management to reduce the tumor size and prevent further growth.

## 6 Follow-up

After six months of compliant glucocorticoid therapy, the patient showed improvement in hormone levels with a decrease in ACTH and androgen levels. Repeat ultrasound demonstrated a slight reduction in the size of the testicular masses, confirming a response to medical treatment. The patient's clinical symptoms stabilized, and he remained under regular follow-up for monitoring tumor size and adrenal function.

## 7 Discussion

Testicular adrenal rest tumors are a known but rare complication in males with congenital adrenal hyperplasia, particularly in those with poorly controlled or untreated disease. Development of TART is quite a common complication in adult male patients with CAH and it is mainly related to poor disease control.^3^. These tumors represent hyperplastic adrenal tissue in the testes due to elevated ACTH levels stimulating ectopic adrenal remnants. Early diagnosis and adequate hormonal control are essential to prevent irreversible testicular damage, which could lead to infertility.^4^. Although biopsy is generally not the first-line diagnostic approach, but it may be performed in rare cases where the diagnosis is uncertain or other testicular tumors need to be excluded^1^^,^^2^. In this case, there is suspicious of malignancy such as seminoma that is why we performed testicular biopsy. These lesions are potentially reversible, as demonstrated by the disappearance/reduction after adjustment of cortisone therapy and by the reduction in plasma ACTH level. Management primarily focuses on optimizing glucocorticoid therapy to reduce ACTH stimulation and suppress tumor growth.^5^.

## 8 Conclusion

This case highlights the importance of long-term adherence to glucocorticoid therapy in patients with congenital adrenal hyperplasia to prevent complications such as testicular adrenal rest tumors. This should have been recognized and evaluated as such without surgical intervention. Biopsy was a reasonable alternative to a trial of medical therapy.

## CRediT authorship contribution statement

**Amir Reza Abedi:** Supervision, Resources, Data curation. **Azade Rakhshan:** Resources, Data curation. **Mohammad Seifi Poor:** Writing – review & editing, Writing – original draft, Methodology, Funding acquisition, Data curation, Conceptualization.
